# Abnormal Prefrontal Functional Connectivity Is Associated with Inflexible Information Processing in Patients with Autism Spectrum Disorder (ASD): An fNIRS Study

**DOI:** 10.3390/biomedicines10051132

**Published:** 2022-05-13

**Authors:** Melody M. Y. Chan, Ming-Chung Chan, Oscar Long-Hin Lai, Karthikeyan Krishnamurthy, Yvonne M. Y. Han

**Affiliations:** 1 Department of Rehabilitation Sciences, The Hong Kong Polytechnic University, Hong Kong, China; 18041801r@connect.polyu.hk (M.M.Y.C.); 18044909r@connect.polyu.hk (M.-C.C.); 19011253g@connect.polyu.hk (O.L.-H.L.); karthikeyan.krishnamurthy@connect.polyu.hk (K.K.); 2 University Research Facility in Behavioral and Systems Neuroscience (UBSN), The Hong Kong Polytechnic University, Hong Kong, China

**Keywords:** autism, cognitive flexibility, WCST, processing speed, functional connectivity, fNIRS

## Abstract

Individuals with autism spectrum disorder (ASD) are characterized by impairments in flexibly acquiring and maintaining new information, as well as in applying learned information for problem solving. However, the neural mechanism underpinning such impairments remains unclear. This study investigated the flexibility in the acquisition and application of visual information in ASD (aged 14–21) when they performed the Wisconsin Card Sorting Test (WCST). Behavioral data including response accuracy and latency, and prefrontal hemodynamic data measured by functional near-infrared spectroscopy (fNIRS), were collected when individuals performed WCST. Canonical general linear model and functional connectivity analyses were performed to examine the prefrontal activation and synchronization patterns, respectively. Results showed that although ASD individuals (*n* = 29) achieved comparable accuracy rates when compared with age- and intelligence quotient (IQ)-matched typically developing (TD; *n* = 26) individuals (F_1,53_ = 3.15, *p* = 0.082), ASD individuals needed significantly more time to acquire and apply WCST card sorting rules (F_1,53_ = 17.92, *p* < 0.001). Moreover, ASD individuals showed significantly lower prefrontal functional connectivity than TD individuals during WCST (F_1,42_ = 9.99, *p* = 0.003). The hypoconnectivity in ASD individuals was highly significant in the right lateral PFC in the acquisition condition (*p* = 0.005) and in the bilateral lateral PFC in the application condition (ps = 0.006). Furthermore, slower WCST reaction time was correlated with lower bilateral lateral PFC functional connectivity only in the application condition (ps = 0.003) but not the acquisition condition. Impairment in information acquisition and application is evident in ASD individuals and is mediated by processing speed, which is associated with lower functional connectivity in the bilateral lateral PFC when these individuals apply learned rules to solve novel problems.

## 1. Introduction

Autism spectrum disorder (ASD) is a pervasive neurodevelopmental disorder that is diagnosed in approximately one in 54 individuals [1]. These individuals are characterized by exhibiting persistent social deficits and restricted, repetitive behaviors that significantly affect daily social and occupational functioning [2]. Individuals with ASD show marked impairment in adjusting behaviors to accommodate different social situations [3], demonstrate strong adherence to inflexible routines, and uncontrollable temper outbursts over trivial changes in the environment [4]. Given that the ability to adjust behaviors to ever-changing daily situations has been shown to be associated with cognitive flexibility [5], some researchers have hypothesized that deficits in cognitive flexibility may underpin the behavioral manifestations of ASD [6,7].

Cognitive flexibility, which comprises cognitive processes needed to acquire and maintain new information and to apply learned information for problem solving, has long been conceptualized as a vital ability for humans to process daily environmental information [5,8,9,10]. The Wisconsin Card Sorting Task (WCST) is a widely used neuropsychological test which assess cognitive flexibility in both healthy and clinical populations [11,12,13]. The test requires participants to sort the response cards correctly with several stimulus cards through feedback given to them based on a rule [14]. WCST can be operationally stratified into substages assessing an individual’s ability to process information, in particular the acquisition and maintenance of new information, as well as the application of learned information [8]. For ASD individuals, WCST is revealed to be the only cognitive flexibility task that has reflected their consistent deficits in this aspect [15]. Specifically, Van Eylen et al. [7] showed that individuals with ASD, when compared to their typically developing (TD) counterparts, tended to produce more perseveration errors and required a longer processing time to produce a response–indexed by increased latency–when the card sorting rules changed. A neurophysiological study conducted by Yeung et al. [16] further showed that abnormal neurophysiology in the frontal cortex of individuals with ASD was associated with reduced accuracy and increased latency in WCST task performance. Information processing speed has been found to be an indicator of how efficiently the brain operates to process information including registering and encoding stimuli, or retrieving existing information [17]. With reports of impaired information processing in ASD [18,19], these studies collectively imply that cognitive flexibility deficits in individuals with ASD, represented by a reduction in WCST task accuracy, are possibly modulated by information processing speed as reflected by an increased latency for task completion; moreover, the impaired performance on the WCST may be associated with abnormal frontal cortex functioning in these individuals.

A previous meta-analysis showed that during both rule acquisition and application stages in the WCST, brain regions within the prefrontal cortex (PFC) are predominantly activated, which includes the bilateral inferior, middle and superior/medial frontal gyri, as well as the anterior cingulate gyrus [20]. Increasing evidence reveals that integrated activities, specifically the neural connectivity among these brain regions, underpin WCST performance [21,22]. Individuals with ASD are known to have frontal lobe abnormalities [16,23] and abnormal neural connectivity [18]. Therefore, given the great dependence on the integrated actions of frontal brain regions needed to complete the WCST, it is reasonable to speculate that altered neural connectivity in the frontal brain regions of these individuals could also be observed during WCST, which is possibly associated with the cognitive flexibility deficits noted in these individuals. Indeed, previous studies have documented aberrant frontal cortex activation in individuals with ASD during various cognitive flexibility tasks [24,25], yet the difference in frontal activation and neural connectivity during WCST between individuals with ASD and their TD counterparts, as well as the relationships between neurophysiological and behavioral parameters, remain elusive.

One of the parameters reflecting neural connectivity is functional connectivity–the Pearson’s correlation between filtered signals recorded from different parts of the brain that reflect the degree of nondirectional synchrony between two brain regions [26]. To measure functional connectivity, an increasing number of studies have utilized functional near-infrared spectroscopy (fNIRS), an optical neuroimaging tool that has been widely applied to study the hemodynamic responses evoked by neuronal activity [27] in the PFC of healthy [28] and clinical populations, such as individuals with ASD [29]. Given that previous evidence has shown that cognitive flexibility deficits in individuals with ASD are associated with abnormal frontal functioning, this study aimed to investigate frontal cortex functioning–in terms of activation and functional connectivity–in individuals with ASD during the WCST. The psychophysiological correlates of impaired WCST performance in individuals with ASD were also explored. We hypothesized that individuals with ASD have an impaired ability to flexibly acquire and apply new information, as reflected by poorer WCST behavioral performance when compared to that of TD individuals. We further hypothesized that the observed behavioral impairment is associated with abnormal prefrontal activation and functional connectivity, as measured by fNIRS.

## 2. Materials and Methods

### 2.1. Participants

**Figure 1:**
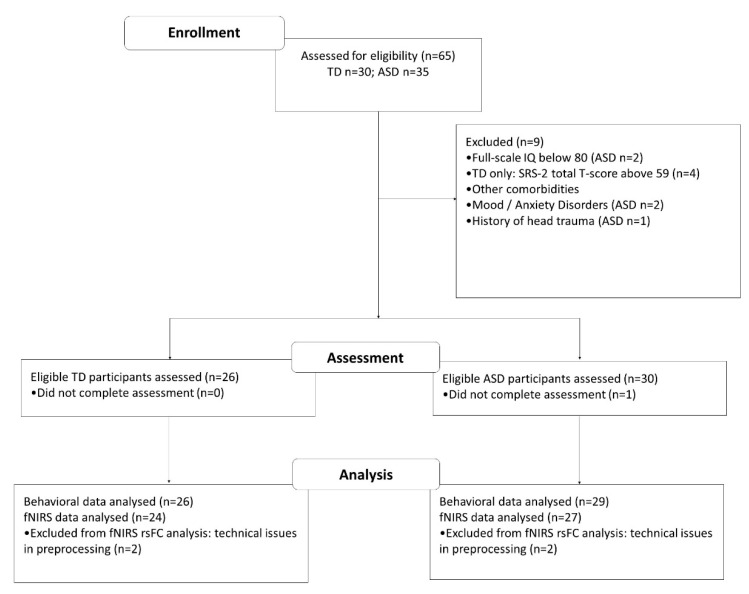
A flowchart showing the participants’ enrollment, assessment, and analysis of this study.

This study was approved by the Human Subjects Ethics Sub-Committee of the Hong Kong Polytechnic University (HSEARS20190319006; approval date: 22 March 2019) and was conducted in accordance with the Declaration of Helsinki. All participants were between 14 and 22 years of age, and achieved a full intelligence quotient (IQ) of ≥80 measured by either the short form of Hong Kong Wechsler Intelligence Scale for Children–fourth edition (WISC-IV-HK:SF; [30]) for participants aged below 16 or the short form of Wechsler Adult intelligence Scale–fourth edition (WAIS-IV-HK:SF; [31]) for those who were aged 16 years or above. Given that previous research has shown that individuals with ASD exhibit sex-dependent differences in nonsocial cognitive domains involving executive function [32], we only included males in our sample. Furthermore, as prior studies have also shown that handedness influences the brain network organization underlying executive functioning [33], we only included right-handed individuals, whose handedness was confirmed by scoring over +80 on the Edinburgh Handedness Inventory short form [34]. The diagnosis of ASD in participants was confirmed by Autism Diagnostic Interview–Revised (ADI-R; [35]). In addition, the social functioning of TD individuals was screened by the second edition of the Social Responsiveness Scale (SRS-2, [36]), with all included TD individuals obtaining a total T-score equal to or below 59, indicating that they had normal daily social functioning. A total of 65 individuals potentially suitable for being included in this study were recruited from multiple sources, including a child psychiatry clinic of a public hospital, a private autism clinic run by allied health professionals, and social media (i.e., Facebook local peer support group, WhatsApp). All of the recruited individuals were assessed for eligibility. Nine individuals were excluded from the participation of this study as they did not fulfill the inclusion criteria listed above. Twenty-nine individuals with ASD and 26 TD individuals aged 14–21 years completed the assessment, and their data were included in the final analysis. The participants’ flow is illustrated in Figure 1.

### 2.2. Procedure and Materials

Before the commencement of the experiment, the procedures and potential risks and benefits of the study were first explained to the participants and their parents; written informed consent was then obtained from all participating parents of the recruited individuals. All children underwent two assessments, including an IQ assessment and a fNIRS measurement. The sequence of assessments was counterbalanced across subjects to minimize order effects. While parents of all children were asked to complete the SRS-2, parents of children with ASD were involved in a structured interview with ADI-R administered after the completion of the SRS-2. The WISC/WAIS-IV-HK:SF and ADI-R were administered by a clinical psychologist who was blinded to the hypothesis of the study. fNIRS measurements were conducted by trained research assistants.

### 2.3. fNIRS Measurement

**Figure 2:**
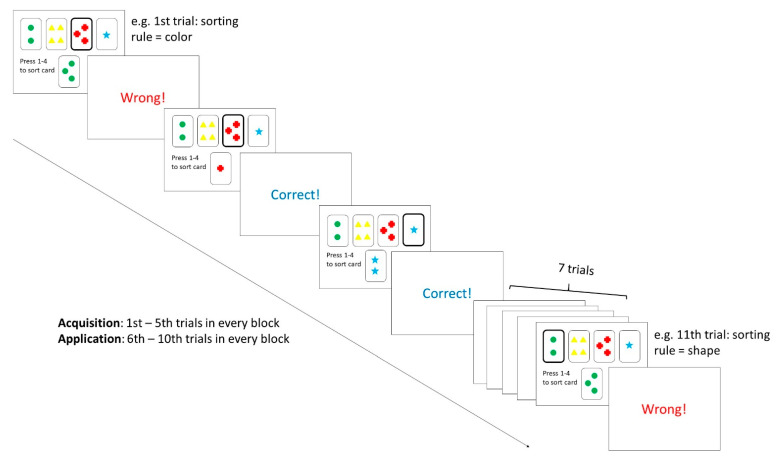
Experimental design. The WCST-64 paradigm consists of six blocks with 10 trials for each block. Participants were asked to sort the stimulus card by a rule (color, shape, number) that was not disclosed to them. The rule changes after every 10 trials and the rule order were counterbalanced among subjects. In every trial, participants were presented with one stimulus card (**below**) and four reference cards ((**top row**); remained unchanged throughout the whole paradigm). The order of stimulus cards was random. Feedback indicating choice accuracy was given immediately after each response was made.

**Figure 3:**
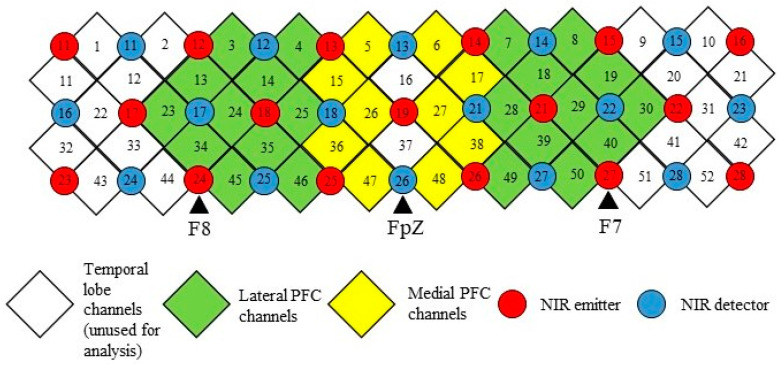
The arrangements of near-infrared channels, emitters, and detectors, as well as the channel groupings.

For each of the participants, fNIRS measurements were taken before and during the WCST. Before the commencement of the WCST paradigm, participants were asked to focus on a fixation cross for 30 s, such that the baseline neural activity could be recorded. A computerized version of the WCST–64 card version [37] was then presented using E-prime 3.0 software (Psychology Software Tools Inc., Sharpsburg, PA, USA). The WCST experimental design was adopted from Vatansever, Menon [8] and is illustrated in Figure 2. A 52-channel fNIRS optical topography system (ETG-4000; Hitachi Medical Co., Tokyo, Japan) was used to measure the changes in brain signals during the task with a sampling rate of 10 Hz. Before wearing the probe set, the 10–20 system [38] landmarks were identified by measuring the head circumference and distance between the inion and nasion, as well as between the left and right auricular. To ensure measurement consistency, this procedure was performed by the same experienced research assistant. The probe was set with thirty-three NIR emitters, and detectors were mounted on a 3 × 11 grid with the emitter-detector distance fixed at 3 cm, which was then worn on a participant’s head using an elastic headband with detector 26 placed at FpZ, emitter 24 placed at the midway between F8, and emitter 27 placed at the midway between F7. Given the WCST task has shown to primarily involve the medial and lateral PFC [20], and to increase the signal-noise ratio of the fNIRS data [29], the channels were grouped into four regions-of-interest (ROI), to represent the medial and lateral PFC in the left and right hemispheres. The arrangements of channels, NIR emitters, and detectors as well as the channel groupings are illustrated in Figure 3. The Montreal Neurological Institute (MNI) coordinates and anatomical labels of each NIRS channel are shown in Table S1.

### 2.4. Data Analysis

*fNIRS preprocessing*. fNIRS data preprocessing was performed using the AnalyzIR toolbox [39] in MATLAB 2019a (The MathWorks, Natick, MA, USA), and the preprocessing pipeline is illustrated below. The light intensity data for each participant collected during baseline and WCST conditions were extracted from the ETG-4000 machine (Hitachi Medical Co., Tokyo, Japan). Saturated or flatlined channels were replaced with high variance noise using *FixSatChans* or *FixFlatChans* modules, respectively. Next, the *Resample* module was adopted to downsample the data to 1 Hz. A downsampling rate of 1 Hz was chosen so as to reduce the cardiac oscillations (around 1 Hz, [40]) present in the optical signals [41,42]. As the new sampling rate (1 Hz) is smaller than the Nyquist frequency of the sampling rate of the original signal (sampled at 10 Hz by ETG-4000 NIRS machine), aliasing–a form of data distortion–could be resulted [43]. To avoid this issue, a polyphase antialiasing filter was applied to remove the distorted components above the Nyquist frequency of the new sampling rate. The resampled data were converted to optical density using the *OpticalDensity* module and then to oxyhemoglobin (HbO), deoxyhemoglobin (HbR), and total hemoglobin (HbT) concentrations using the modified Beer–Lambert Law. For statistical analysis, only HbO data were used in this study given that HbO has been found to be a more sensitive measure than HbR in detecting task-related neural changes in patients with neurological disorders, including ASD [44].

*fNIRS first-level analysis*. To estimate the differences in fNIRS activation patterns between the two experimental (i.e., acquisition/application) conditions and the baseline (i.e., focusing on a fixation cross) condition while also controlling for the type I errors caused by slow systemic physiology and motion artifacts, a canonical general linear model (GLM) with an autoregressive prewhitening approach using iteratively reweighted least-squares (AR-IRLS; [45]) was performed for the HbO data point of each of the participants. The GLM yielded a regression coefficient (β) for each channel in each of the experimental conditions. The β-values were summed and averaged within each ROI (i.e., the left medial PFC, right medial PFC, left lateral PFC and right lateral PFC) for acquisition and application conditions. These averaged values were used in the second-level fNIRS activation analysis.

To estimate the differences in fNIRS functional connectivity between experimental (i.e., acquisition/application) and baseline (i.e., focusing on a fixation cross) conditions, GLM with AR-IRLS followed by robust regression [46] were conducted for the HbO data of each of the participants. Although AR-IRLS has been shown to effectively reduce type-I errors in fNIRS activation analyses, shifting-type motion artifacts–which usually result from an individual’s abrupt and excessive movement causing the entire probe set to shift–could still appear as extreme statistical outliers [45]. This effect could yield high but spurious correlations that influence the interpretation of functional connectivity data [46]. Robust regression was hence adopted to reduce the effect of these statistical outliers on data analysis by assigning less weight to the outliers to normalize the noise distributions. A Z-transformed correlation coefficient (Z) was calculated for each of the possible channel pairs, and the Z values were averaged within each region of interest (i.e., the left medial PFC, right medial PFC, left lateral PFC, and right lateral PFC) for both acquisition and application conditions. These averaged values were used in the second-level fNIRS functional connectivity analysis.

*Second-level analysis*. Group-level analyses for behavioral and fNIRS data were performed using IBM SPSS Statistics Version 26.0 (IBM Corp, Armonk, NY, USA). The normality of the data was first checked with the Shapiro–Wilk test. To examine whether the individuals in the TD and ASD groups were matched, independent sample t tests (or Mann–Whitney tests for nonnormal data) were performed for age, IQ, and SRS-2 data. To test for the difference in WCST performance between individuals in the two groups during the acquisition and application stages, accuracy in terms of correct percentage and reaction time (ms) were analyzed with 2 (group) × 2 (category) mixed ANOVA. To investigate the difference in prefrontal fNIRS activation patterns in the medial/lateral PFC of the left/right hemisphere between the two groups during different conditions (i.e., acquisition/application) in the WCST, a 2 (group) × 2 (category) × 2 (region) × 2 (hemisphere) mixed ANOVA was performed with the averaged β-values of the medial/lateral PFC of each hemisphere. To investigate the difference in prefrontal fNIRS functional connectivity within the medial/lateral PFC of the left/right hemisphere between the two groups during different conditions (i.e., acquisition/application) in the WCST, a 2 (group) × 2 (category) × 2 (region) × 2 (hemisphere) mixed ANOVA was performed with the averaged Z values of the medial/lateral PFC of each hemisphere. Post hoc t tests were performed when significant interaction or main effects were found for the above analyses. To explore the relationship between brain hemodynamic changes and behavioral performance, Spearman’s rank order correlational analyses (two-tailed) were conducted for parameters that were determined to be significant in the group comparison at both the whole-group and subgroup levels. Given prefrontal maturation is heavily related to age in adolescents and young adults, we also performed Spearman’s rank order correlational analyses (two-tailed) to explore the relationships between age, WCST behavioral performance, and fNIRS measures. Regarding fNIRS activation and functional connectivity analyses, the significance level (alpha = 0.05) with Bonferroni corrections for two experimental conditions and four ROIs is kept at *p* = 0.05/8 = 0.006. Bonferroni corrections were applied to all post hoc and correlational analyses with an alpha level kept at *p* = 0.05 unless otherwise specified.

## 3. Results

### 3.1. Demographic Details

**Figure 4:**
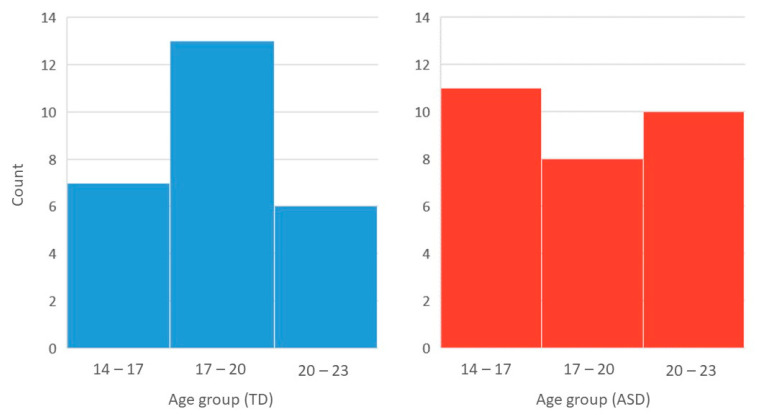
Histograms showing the age distributions of the TD (**left**) and ASD (**right**) group.

The demographic details of the participants are listed in Table 1. Independent sample *t* tests revealed that individuals in the ASD and TD groups were matched for full-scale IQ (*p* = 0.293) and age (*p* = 0.259), with the age distributions of each group shown in Figure 4. The ADI-R domain scores were all above the cutoff for our ASD sample, with this group of individuals also showing markedly impaired current social functioning when compared to their TD counterparts, indicated by significantly higher SRS-2 total scores (*p* < 0.001).

### 3.2. WCST Behavioral Performance

The behavioral performance results of the WCST are listed in Table 2. In terms of performance accuracy, a 2 × 2 mixed ANOVA showed a nonsignificant group*condition interaction effect (F_1,53_ = 0.342, *p* = 0.561) with a nonsignificant main effect of group (F_1,53_ = 3.15, *p* = 0.082). Regarding reaction time, a 2 × 2 mixed ANOVA showed a highly significant main effect of group (F_1,53_ = 17.92, *p* < 0.001), and post hoc independent-sample t tests indicated that individuals with ASD took significantly longer to respond during both the acquisition (*p* < 0.001) and application conditions. The group*condition interaction effect was nonsignificant (F_1,53_ = 0.026, *p* = 0.872).

### 3.3. fNIRS Prefrontal Activation during the WCST

A 4-way mixed ANOVA revealed a nonsignificant group*condition*region*hemisphere interaction effect (F_1,49_ = 0.315, *p* = 0.577) and a nonsignificant main effect of group (F_1,49_ = 0.599, *p* = 0.443) for prefrontal activation during the WCST. The descriptive statistics are shown in Table 3.

### 3.4. fNIRS Prefrontal Functional Connectivity during the WCST

Regarding intrahemispheric functional connectivity within the medial and lateral PFC (Table 4), a 4-way mixed ANOVA revealed a highly significant main effect of group (F_1,49_ = 9.99, *p* = 0.003) and a nonsignificant group*condition*region*hemisphere interaction (F_1,49_ = 1.82, *p* = 0.185). In the acquisition condition, individuals with ASD exhibited lower functional connectivity in the right lateral PFC (*p* = 0.005), while in the application condition, the bilateral PFC exhibited lower functional connectivity (right: *p* = 0.006; left: *p* = 0.006).

### 3.5. Brain-Behavior Relationships

**Figure 5:**
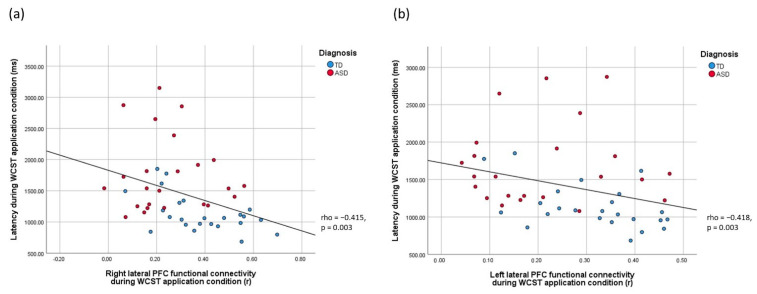
Scatter plots showing the brain-behavior relationships between WCST response latency during WCST application condition and lateral PFC functional connectivity in the (**a**) right and (**b**) left hemispheres, respectively. Red dots represent data from the ASD group, blue dots represent data from the TD group.

The relationships between reaction time in the WCST, right PFC functional connectivity during the acquisition condition, and bilateral PFC functional connectivity during the application condition were examined. Correlations between WCST reaction time during the acquisition condition and fNIRS measures at the whole-group level were significant at the *p* = 0.05 (uncorrected) level but did not survive Bonferroni corrections (rhos > −0.362, ps > 0.01, uncorrected; Table 5). In turn, highly significant negative correlations between reaction time in the WCST in the application condition and bilateral lateral PFC functional connectivity were found (rhos = < −0.415, ps = 0.003, Bonferroni-corrected, Table 5). For the TD subgroup, the negative correlation between reaction time in the WCST in the application condition and right lateral PFC functional connectivity remained to be significant with Bonferroni corrections (rho = −0.529, *p* = 0.008; Table S2). For the ASD subgroup, all correlations between WCST reaction time and fNIRS measures were nonsignificant (ps > 0.05, Table S2). Visual inspection of individual data (Figure 5, Bonferroni-corrected) revealed that the heterogeneity in WCST performance was greater among ASD than TD individuals. Regarding the relationship between age, behavioral, and optical imaging measures, all correlations were nonsignificant (ps > 0.05, Bonferroni-corrected) at the whole-group and subgroup levels (Table S3).

## 4. Discussion

This study aimed to investigate the neuropsychological and neurophysiological functioning of individuals with ASD when they performed a well-known cognitive flexibility task, the WCST. Behavioral and fNIRS data from 29 individuals with ASD were compared with those from 26 age- and IQ-matched TD counterparts. The results showed that although individuals with ASD achieved comparable accuracy rates when compared with those of TD individuals, individuals with ASD needed significantly more time to acquire and apply the WCST card sorting rules. Moreover, individuals with ASD showed significantly lower functional connectivity in the right lateral PFC during the acquisition condition and in the bilateral lateral PFC during the application condition. Furthermore, slower WCST reaction time was correlated with lower bilateral lateral PFC functional connectivity only in the application condition but not in the acquisition condition.

Consistent with previous studies, individuals with ASD were found to exhibit impairments in all cognitive processes supporting cognitive flexibility, namely, information acquisition and maintenance, as well as information application for problem solving. For instance, Young et al. [47] determined that children with ASD demonstrated a reduction in information acquisition behavior when they were asked to learn novel actions in response to different social scenarios. For information maintenance, Miller et al. [48] showed that children with ASD exhibited marked difficulties in maintaining learned rules for task completion, of which such impairment was positively correlated with increased severity in restricted, repetitive behaviors. Regarding information application for problem solving, Yeung et al. [29] showed that individuals with ASD exhibited marked impairment when they were asked to produce learned words that were relevant to a semantic rule. In particular, as shown by our results, these individuals took significantly more time to yield comparable performance accuracy with their peers, which was also consistent with previous findings showing that information application deficits in individuals with ASD reflected by other executive functioning tasks (e.g., verbal fluency tasks) were only revealed when there was limited time [29]–not ample time [49]–given for task completion. Our results imply that the impairment of cognitive flexibility subprocesses in individuals with ASD is mediated by information processing speed, providing evidence, alongside a number of previous studies (e.g., Haigh et al. [50], Eack et al. [51], Faja et al. [52]), to support the notion that there is a fundamental impairment in speed that affects information processing in individuals with ASD, such that they exhibit abnormalities, especially when demand for information processing is high (e.g., complex behavior including social communication and interaction).

Previous studies have shown that information processing efficiency is underpinned by the strength of functional connectivity between regions within and between functional brain networks [53]. For example, visual information processing speed has been shown to be associated with functional connectivity within the cingulo-opercular [54] and right frontoparietal networks [55], as well as between the cingulo-opercular and the occipital network and between the right frontoparietal network and the occipital network [56]. Individuals with impairments in information processing speed, such as in ASD, are postulated to exhibit aberrant functional connectivity [57]. Indeed, many previous studies have documented altered functional connectivity in individuals with ASD, both during the resting state [18,58] and during many neuropsychological tests requiring complex information processing, including working memory [59,60], face recognition [61], and mentalizing tests [62]. Consistent with these studies, we provided new evidence to support aberrant functional connectivity in individuals with ASD during demanding cognitive tasks. In particular, we showed that individuals with ASD exhibited PFC hypoconnectivity during the WCST. Moreover, we demonstrated that the right lateral PFC showed hypoconnectivity when participants acquired a new rule through trial and error, and the bilateral lateral PFC showed hypoconnectivity when participants were asked to apply the learned rules for problem solving. The result of the condition-independent right lateral PFC hypoconnectivity during WCST in individuals with ASD found in our study is consistent with a recent large-scale lesion study showing that lesions in the right lateral PFC underlie the impaired performance on the WCST [63]. We further showed that, in the context of ASD, slower information processing speed during the WCST task is correlated with lower functional connectivity in the right lateral PFC, particularly when these individuals are asked to apply rules for problem solving. These results collectively imply that reduced right lateral PFC functional connectivity is the neural correlate for speed-mediated cognitive flexibility deficits in individuals with ASD, which particularly affects the application of rules for solving novel problems and manifests in daily situations, such as social communication [64]. Interestingly, we also observed hypoconnectivity in the left, on top of the right, and the lateral PFC in individuals with ASD specifically during the rule application condition, which was also highly correlated with WCST behavioral performance. This domain-specific left lateral PFC impairment might be attributed to the greater extent of left hemisphere abnormalities in individuals with ASD reported in previous studies [65,66]. Although abnormal PFC brain activation in individuals with ASD during cognitive flexibility tasks was shown in previous neuroimaging studies [24,25], we showed nonsignificant brain activation between individuals with ASD and TD individuals during the WCST. The nonsignificant difference between the two groups might be attributed to the large standard deviations in beta values representing brain activation in individuals in both groups, which might possibly be because different brain regions were recruited when participants employ different cognitive strategies for WCST task completion [67].

In this study, we showed that information processing speed mediates cognitive flexibility task performance in individuals with ASD, which is associated with lower functional connectivity in the right lateral PFC. Given these findings, it is reasonable to postulate that treatments that enhance functional connectivity in this region can promote information processing in individuals with ASD, hence alleviating their cognitive flexibility deficits and the associated social and behavioral impairments. Transcranial direct current stimulation (tDCS), a noninvasive neuromodulation technique, might be a potentially promising candidate treatment for these individuals. A previous meta-analysis showed that prefrontal anodal tDCS could enhance functional connectivity in the right anterior cingulate cortex in humans [68]. In addition, multiple studies conducted in healthy [69] as well as clinical [70] populations have shown that tDCS can enhance individuals’ information processing speed. Thus, the effectiveness of anodal tDCS over the right lateral PFC may be a possible direction of future research.

### Limitations

This study provided important insights into the neuropsychological and neurophysiological functioning of individuals with ASD when they performed a well-established cognitive flexibility task. However, several study limitations should be noted for cautious interpretation of the data. Although multiple neuropsychological and neuroimaging studies have suggested that abnormal PFC functioning underpins cognitive flexibility deficits in individuals with ASD [16,24,25], cognitive flexibility may involve the functioning of brain regions beyond the PFC, which includes the parietal and occipital cortices [20,71]. As we adopted a PFC probe set for this fNIRS study, we only know how PFC functional connectivity contributes to WCST deficits in individuals with ASD. The functional connectivity abnormalities in individuals with ASD in brain regions beyond the PFC, as well as between the PFC and other cortices, should be examined in future studies to give us a more comprehensive understanding regarding the functional connectivity deficits and the associated cognitive flexibility deficits in these individuals. In addition, regarding the resampling of fNIRS data, we have chosen the downsampling rate of 1 Hz, which is a frequently adopted practice in previously published optical imaging studies to reduce physiological noises at higher frequencies [40,41,42]. Yet, a 1 Hz sampling rate will limit the information in the signal to be a maximum of 0.5 Hz, which reduces the temporal resolution of the data. Future studies may explore the possibilities of using a different downsampling rate (e.g., 2 Hz). Furthermore, although the data analysis method used in this study–the AR-IRLS algorithm followed by robust regression–has shown to be effective in reducing type-I errors caused by excessive motions commonly seen that affects the data quality of neuroimaging studies involving ASD individuals, the use of other functional connectivity analysis methods involving active tasks, such as psychophysiological interaction (PPI) analysis, which allows the examination of changes in functional connectivity in different experimental conditions [72], might be explored in future fNIRS studies. Last but not least, the subject-level covariance might be underestimated in our fNIRS activation and functional connectivity group-level analyses. Future studies might consider taking the covariance into account by analyzing the data with the AnalyzIR toolbox linear mixed-effects model pipeline.

## 5. Conclusions

This study investigated the neuropsychological and prefrontal neurophysiological functioning of ASD when they acquire and apply new information during a well-established cognitive flexibility task, WCST. Behavioral and fNIRS data collected during WCST from 29 ASD individuals aged 14–21 were compared with those from 26 IQ- and age-matched TD individuals. The results revealed impairment in information acquisition and application in individuals with ASD; this impairment was mediated by processing speed. Furthermore, a lower functional connectivity in the bilateral lateral PFC was found to be associated with slower processing speed when individuals with ASD were asked to apply learned rules to solve novel problems. Future studies might consider investigating the neural correlates of WCST performance beyond the PFC, as well as the effectiveness of neuromodulation techniques on enhancing lateral PFC functional connectivity for improving information processing efficiency and associated social communication deficits in these individuals.

## Figures and Tables

**Table 1:** Participants’ demographic information.

Parameters	Group				
	ASD	TD	*t*	*df*	*p*
**Mean chronological age in years (S.D.)**	17.75 (2.31)	18.47 (2.36)	1.14	53	0.259
**Mean full Scale IQ (S.D.)**	97.93 (15.98)	101.73 (8.95)	1.07	53	0.293
**Mean SRS-2 total score (S.D.)**	98.86 (25.15)	55.60 (24.29)	−6.00 ***	53	<0.001
**Mean ADI-R domain scores (S.D.)**					
Social interaction	19.30 (3.60)	N/A	N/A		
Communication	14.67 (4.29)				
Restricted, repetitive behavior	3.30 (1.92)				

Note: All participants (ASD *n* = 29, TD *n* = 26) were right-handed and male; SRS: Social Responsiveness Scale-2; ADI-R: Autism Diagnostic Interview-Revised; *** *p* < 0.001.

**Table 2:** WCST behavioral performance.

Parameters	Group				
	ASD	TD	*t*	*df*	*p*
**Accuracy (% of correct responses)**					
Acquisition	55.17 (12.07)	60.03 (12.63)	1.46	53	0.151
Application	76.97 (14.47)	83.77 (15.20)	1.70	53	0.095
**Reaction time (ms)**					
Acquisition	1882.33 (581.46)	1346.39 (394.43)	3.95 ***	53	<0.001
Application	1647.53 (597.26)	1123.10 (291.22)	4.20 ***	53	<0.001

Note: *** *p*< 0.001 (Bonferroni-corrected).

**Table 3:** fNIRS prefrontal activation during WCST in ASD and TD individuals (Beta values).

	Right Hemisphere		Left Hemisphere	
	Group		Group	
	ASD	TD	ASD	TD
**Condition 1: Acquisition**				
**Medial**	1.58 (40.83)	7.86 (38.89)	9.85 (34.54)	7.97 (40.32)
**Lateral**	1.60 (35.96)	16.35 (35.88)	7.86 (36.96)	8.40 (46.22)
**Condition 2: Application**				
**Medial**	2.40 (40.50)	8.27 (39.67)	7.96 (37.41)	6.22 (40.67)
**Lateral**	−0.24 (37.33)	16.00 (35.66)	8.21 (38.23)	6.54 (45.37)

Note: *n* = 51 (2 fNIRS data from the TD group and 2 data from ASD group were unable to be preprocessed during technical issues).

**Table 4:** fNIRS prefrontal functional connectivity during WCST in ASD and TD individuals (*r*).

	Right Hemisphere					Left Hemisphere				
	Group					Group				
	ASD	TD	*t*	*df*	*p*	ASD	TD	*t*	*df*	*p*
**Condition 1: Acquisition**										
**Medial**	0.22 (.15)	0.31 (.17)	2.04	49	0.047	0.19 (0.09)	0.24 (0.10)	1.95	49	.057
**Lateral**	0.21 (.17)	0.34 (.17)	2.91 **	49	0.005	0.21 (0.13)	0.30 (0.12)	2.69	49	0.010
**Condition 2: Application**										
**Medial**	0.22 (.14)	0.34 (.18)	2.56	49	0.014	0.20 (0.08)	0.25 (0.12)	1.86	49	0.069
**Lateral**	0.25 (.16)	0.38 (.16)	2.89 **	49	0.006	0.21 (0.13)	0.32 (0.11)	2.89 **	49	0.006

Note: *n* = 51 (Two fNIRS data from the TD group and two data from ASD group were unable to be preprocessed during technical issues). ** *p* < 0.0063 (Bonferroni-corrected).

**Table 5:** Correlation table between WCST reaction time and PFC FC parameters (whole-group analysis).

Parameters	R_lPFC_Acquisition	R_lPFC_Application	L_lPFC_Application
**RT_acquisition**	−0.348	−0.362	−0.344
**RT_application**	−0.353	−0.415 **	−0.418 **

*Note: n* = 51 (listwise)*;* ** *p* < 0.0083 (Bonferroni-corrected).

## Data Availability

The data presented in this study are available on reasonable request from the corresponding author.

## Supplementary Materials

The following supporting information can be downloaded at: https://www.mdpi.com/article/10.3390/biomedicines10051132/s1, Table S1: The MNI coordinates and anatomical labels of each NIRS channel, Table S2: Correlation table showing the relationship between age, reaction time measures and fNIRS functional connectivity measures, Table S3: Correlation tables between WCST reaction time and PFC FC parameters (subgroup analyses).

## References

[1] Maenner M.J., Shaw K.A., Baio J. (2020). Prevalence of autism spectrum disorder among children aged 8 years—Autism and developmental disabilities monitoring network, 11 sites, United States, 2016. MMWR Surveill. Summ..

[2] APA (2013). Diagnostic and Statistical Manual of Mental Disorders: DSM-5™.

[3] Russell G., Golding J., Norwich B., Emond A., Ford T., Steer C. (2012). Social and behavioural outcomes in children diagnosed with autism spectrum disorders: A longitudinal cohort study. J. Child Psychol. Psychiatry.

[4] Han Y.M.-Y., Chan A.S.-Y. (2018). Neural Basis of Learning Issues in Children with Autism: A Bridge to Remediation Planning. Routledge International Handbook of Schools and Schooling in Asia.

[5] Duncan J. (2010). The multiple-demand (MD) system of the primate brain: Mental programs for intelligent behaviour. Trends Cogn. Sci..

[6] Memari A.H., Ziaee V., Shayestehfar M., Ghanouni P., Mansournia M.A., Moshayedi P. (2013). Cognitive flexibility impairments in children with autism spectrum disorders: Links to age, gender and child outcomes. Res. Dev. Disabil..

[7] Van Eylen L., Boets B., Steyaert J., Evers K., Wagemans J., Noens I. (2011). Cognitive flexibility in autism spectrum disorder: Explaining the inconsistencies?. Res. Autism Spectr. Disord..

[8] Vatansever D., Menon D.K., Stamatakis E.A. (2017). Default mode contributions to automated information processing. Proc. Natl. Acad. Sci. USA.

[9] Armbruster-Genç D.J.N., Ueltzhöffer K., Fiebach C.J. (2016). Brain Signal Variability Differentially Affects Cognitive Flexibility and Cognitive Stability. J. Neurosci..

[10] Kehagia A.A., Murray G., Robbins T. (2010). Learning and cognitive flexibility: Frontostriatal function and monoaminergic modulation. Curr. Opin. Neurobiol..

[11] Miles S., Howlett C.A., Berryman C., Nedeljkovic M., Moseley G.L., Phillipou A. (2021). Considerations for using the Wisconsin Card Sorting Test to assess cognitive flexibility. Behav. Res. Methods.

[12] Westwood H., Stahl D., Mandy W., Tchanturia K. (2016). The set-shifting profiles of anorexia nervosa and autism spectrum disorder using the Wisconsin Card Sorting Test: A systematic review and meta-analysis. Psychol. Med..

[13] Uddin L.Q. (2021). Cognitive and behavioural flexibility: Neural mechanisms and clinical considerations. Nat. Rev. Neurosci..

[14] Coulacoglou C., Saklofske D.H., Coulacoglou C., Saklofske D.H. (2017). Chapter 5-Executive Function, Theory of Mind, and Adaptive Behavior. Psychometrics and Psychological Assessment.

[15] Landry O., Al-Taie S. (2016). A Meta-analysis of the Wisconsin Card Sort Task in Autism. J. Autism Dev. Disord..

[16] Yeung M.K., Han Y.M.Y., Sze S.L., Chan A.S. (2016). Abnormal frontal theta oscillations underlie the cognitive flexibility deficits in children with high-functioning autism spectrum disorders. Neuropsychology.

[17] Neubauer A.C., Fink A. (2003). Fluid intelligence and neural efficiency: Effects of task complexity and sex. Pers. Individ. Differ..

[18] Han Y.M., Chan A. (2017). Disordered cortical connectivity underlies the executive function deficits in children with autism spectrum disorders. Res. Dev. Disabil..

[19] Williams D.L., Goldstein G., Minshew N.J. (2006). Neuropsychologic Functioning in Children with Autism: Further Evidence for Disordered Complex Information-Processing. Child Neuropsychol..

[20] Buchsbaum B.R., Greer S., Chang W.-L., Berman K.F. (2005). Meta-analysis of neuroimaging studies of the Wisconsin Card-Sorting task and component processes. Hum. Brain Mapp..

[21] Carrillo-De-La-Peña M., Garcia-Larrea L. (2007). Right frontal event related EEG coherence (ERCoh) differentiates good from bad performers of the Wisconsin Card Sorting Test (WCST). Neurophysiol. Clin. Neurophysiol..

[22] Zhang C., Yang H., Qin W., Liu C., Qi Z., Chen N., Li K. (2017). Characteristics of resting-state functional connectivity in intractable unilateral temporal lobe epilepsy patients with impaired executive control function. Front. Hum. Neurosci..

[23] Teffer K., Semendeferi K. (2012). Human prefrontal cortex: Evolution, development, and pathology. Prog. Brain Res..

[24] Gilbert S.J., Bird G., Brindley R., Frith C., Burgess P.W. (2008). Atypical recruitment of medial prefrontal cortex in autism spectrum disorders: An fMRI study of two executive function tasks. Neuropsychologia.

[25] Yerys B.E., Antezana L., Weinblatt R., Jankowski K., Strang J., Vaidya C.J., Schultz R.T., Gaillard W.D., Kenworthy L. (2015). Neural Correlates of Set-Shifting in Children With Autism. Autism Res..

[26] Friston K.J. (2011). Functional and Effective Connectivity: A Review. Brain Connect..

[27] Villringer A., Chance B. (1997). Non-invasive optical spectroscopy and imaging of human brain function. Trends Neurosci..

[28] Huang C.-J., Chou P.-H., Wei H.-L., Sun C.-W., Lin W.-H. (2015). Functional Connectivity During Phonemic and Semantic Verbal Fluency Test: A Multichannel Near Infrared Spectroscopy Study. IEEE J. Sel. Top. Quantum Electron..

[29] Yeung M.K., Lee T.L., Chan A.S. (2019). Frontal lobe dysfunction underlies the differential word retrieval impairment in adolescents with high-functioning autism. Autism Res..

[30] Wechsler D., Kodama H. (2012). Wechsler Intelligence Scale for Children.

[31] Wechsler D. (2008). Wechsler Adult Intelligence Scale, Fourth Edition (WAIS-IV).

[32] Lai M.-C., Lombardo M., Ruigrok A., Chakrabarti B., Wheelwright S., Auyeung B., Allison C., Baron-Cohen S. (2012). MRC AIMS Consortium Cognition in Males and Females with Autism: Similarities and Differences. PLoS ONE.

[33] Gao Q., Wang J., Yu C., Chen H. (2015). Effect of handedness on brain activity patterns and effective connectivity network during the semantic task of Chinese characters. Sci. Rep..

[34] Oldfield R.C. (1971). The assessment and analysis of handedness: The Edinburgh inventory. Neuropsychologia.

[35] Lord C., Rutter M., Le Couteur A. (1994). Autism Diagnostic Interview-Revised: A revised version of a diagnostic interview for caregivers of individuals with possible pervasive developmental disorders. J. Autism Dev. Disord..

[36] Constantino J.N., Gruber C.P. (2012). Social Responsiveness Scale: SRS-2.

[37] Kongs S.K., Thompson L.L., Iverson G.L., Heaton R.K. (2000). Wisconsin Card Sorting Test-, 64 Card Version: WCST-64.

[38] Jasper H.H. (1958). The ten-twenty electrode system of the International Federation. Electroencephalogr. Clin. Neurophysiol..

[39] Santosa H., Zhai X., Fishburn F., Huppert T. (2018). The NIRS Brain AnalyzIR Toolbox. Algorithms.

[40] Pinti P., Aichelburg C., Gilbert S., Hamilton A., Hirsch J., Burgess P., Tachtsidis I. (2018). A Review on the Use of Wearable Functional Near-Infrared Spectroscopy in Naturalistic Environments. Jpn. Psychol. Res..

[41] Medvedev A.V. (2014). Does the resting state connectivity have hemispheric asymmetry? A near-infrared spectroscopy study. NeuroImage.

[42] Perlman S.B., Luna B., Hein T.C., Huppert T.J. (2014). fNIRS evidence of prefrontal regulation of frustration in early childhood. NeuroImage.

[43] Pinti P., Scholkmann F., Hamilton A., Burgess P., Tachtsidis I. (2019). Current Status and Issues Regarding Pre-processing of fNIRS Neuroimaging Data: An Investigation of Diverse Signal Filtering Methods Within a General Linear Model Framework. Front. Hum. Neurosci..

[44] Yeung M.K., Lin J. (2021). Probing depression, schizophrenia, and other psychiatric disorders using fNIRS and the verbal fluency test: A systematic review and meta-analysis. J. Psychiatr. Res..

[45] Barker J.W., Aarabi A., Huppert T.J. (2013). Autoregressive model based algorithm for correcting motion and serially correlated errors in fNIRS. Biomed. Opt. Express.

[46] Santosa H., Aarabi A., Perlman S.B., Huppert T.J. (2017). Characterization and correction of the false-discovery rates in resting state connectivity using functional near-infrared spectroscopy. J. Biomed. Opt..

[47] Young N., Hudry K., Trembath D., Vivanti G. (2016). Children With Autism Show Reduced Information Seeking When Learning New Tasks. Am. J. Intellect. Dev. Disabil..

[48] Miller H.L., Ragozzino M., Cook E.H., Sweeney J.A., Mosconi M.W. (2015). Cognitive Set Shifting Deficits and Their Relationship to Repetitive Behaviors in Autism Spectrum Disorder. J. Autism Dev. Disord..

[49] Ehlen F., Roepke S., Klostermann F., Baskow I., Geise P., Belica C., Tiedt H.O., Behnia B. (2020). Small Semantic Networks in Individuals with Autism Spectrum Disorder Without Intellectual Impairment: A Verbal Fluency Approach. J. Autism Dev. Disord..

[50] Haigh S.M., Walsh J.A., Mazefsky C.A., Minshew N.J., Eack S.M. (2018). Processing Speed is Impaired in Adults with Autism Spectrum Disorder, and Relates to Social Communication Abilities. J. Autism Dev. Disord..

[51] Eack S.M., Bahorik A.L., McKnight S.A., Hogarty S.S., Greenwald D.P., Newhill C.E., Phillips M.L., Keshavan M.S., Minshew N.J. (2013). Commonalities in social and non-social cognitive impairments in adults with autism spectrum disorder and schizophrenia. Schizophr. Res..

[52] Faja S., Webb S.J., Merkle K., Aylward E., Dawson G. (2009). Brief Report: Face Configuration Accuracy and Processing Speed Among Adults with High-Functioning Autism Spectrum Disorders. J. Autism Dev. Disord..

[53] Silva PH R.D., Secchinato K.F., Rondinoni C., Leoni R.F. (2020). Brain Structural–Functional Connectivity Relationship Underlying the Information Processing Speed. Brain Connect..

[54] Ruiz-Rizzo A.L., Sorg C., Napiórkowski N., Neitzel J., Menegaux A., Müller H.J., Vangkilde S., Finke K. (2019). Decreased cingulo-opercular network functional connectivity mediates the impact of aging on visual processing speed. Neurobiol. Aging.

[55] Haupt M., Ruiz-Rizzo A.L., Sorg C., Finke K. (2020). Right-lateralized fronto-parietal network and phasic alertness in healthy aging. Sci. Rep..

[56] Küchenhoff S., Sorg C., Schneider S.C., Kohl O., Müller H.J., Napiórkowski N., Menegaux A., Finke K., Ruiz-Rizzo A.L. (2021). Visual processing speed is linked to functional connectivity between right frontoparietal and visual networks. Eur. J. Neurosci..

[57] Minshew N.J., Williams D.L., McFadden K. (2008). Information Processing, Neural Connectivity, and Neuronal Organization, in Autism.

[58] Fishman I., Keown C.L., Lincoln A.J., Pineda J.A., Müller R.-A. (2014). Atypical Cross Talk Between Mentalizing and Mirror Neuron Networks in Autism Spectrum Disorder. JAMA Psychiatry.

[59] Koshino H., Carpenter P.A., Minshew N.J., Cherkassky V.L., Keller T.A., Just M.A. (2005). Functional connectivity in an fMRI working memory task in high-functioning autism. NeuroImage.

[60] Krishnamurthy K., Yeung M.K., Chan A.S., Han Y.M.Y. (2020). Effortful Control and Prefrontal Cortex Functioning in Children with Autism Spectrum Disorder: An fNIRS Study. Brain Sci..

[61] Koshino H., Kana R.K., Keller T.A., Cherkassky V.L., Minshew N.J., Just M.A. (2008). fMRI Investigation of Working Memory for Faces in Autism: Visual Coding and Underconnectivity with Frontal Areas. Cereb. Cortex.

[62] Cole E.J., Barraclough N.E., Andrews T.J. (2019). Reduced connectivity between mentalizing and mirror systems in autism spectrum condition. Neuropsychologia.

[63] Gläscher J., Adolphs R., Tranel D. (2019). Model-based lesion mapping of cognitive control using the Wisconsin Card Sorting Test. Nat. Commun..

[64] Williams D.L., Mazefsky C.A., Walker J.D., Minshew N.J., Goldstein G. (2014). Associations Between Conceptual Reasoning, Problem Solving, and Adaptive Ability in High-functioning Autism. J. Autism Dev. Disord..

[65] D’Cruz A.-M., Mosconi M.W., Steele S., Rubin L.H., Luna B., Minshew N., Sweeney J.A. (2009). Lateralized Response Timing Deficits in Autism. Biol. Psychiatry.

[66] Rinehart N.J., Bradshaw J.L., Brereton A.V., Tonge B.J. (2002). Lateralization in individuals with high-functioning autism and Asperger’s disorder: A frontostriatal model. J. Autism Dev. Disord..

[67] Müller V.I., Langner R., Cieslik E.C., Rottschy C., Eickhoff S.B. (2015). Interindividual differences in cognitive flexibility: Influence of gray matter volume, functional connectivity and trait impulsivity. Anat. Embryol..

[68] Chan M.M., Yau S.S., Han Y.M. (2021). The neurobiology of prefrontal transcranial direct current stimulation (tDCS) in promoting brain plasticity: A systematic review and meta-analyses of human and rodent studies. Neurosci. Biobehav. Rev..

[69] Plewnia C., Schroeder P., Kunze R., Faehling F., Wolkenstein L. (2015). Keep Calm and Carry On: Improved Frustration Tolerance and Processing Speed by Transcranial Direct Current Stimulation (tDCS). PLoS ONE.

[70] Gögler N., Willacker L., Funk J., Strube W., Langgartner S., Napiórkowski N., Hasan A., Finke K. (2017). Single-session transcranial direct current stimulation induces enduring enhancement of visual processing speed in patients with major depression. Eur. Arch. Psychiatry Clin. Neurosci..

[71] Fedorenko E., Duncan J., Kanwisher N. (2013). Broad domain generality in focal regions of frontal and parietal cortex. Proc. Natl. Acad. Sci. USA.

[72] Harrison T.M., McLaren D.G., Moody T.D., Feusner J.D., Bookheimer S.Y. (2017). Generalized Psychophysiological Interaction (PPI) analysis of memory related connectivity in individuals at genetic risk for alzheimer’s disease. JoVE J. Vis. Exp..

