# Supplementary material for: Abnormal Prefrontal Functional Connectivity Is Associated with Inflexible Information Processing in Patients with Autism Spectrum Disorder (ASD): An fNIRS Study
Source: Biomedicines. 2022 May 13;10(5):1132. doi: 10.3390/biomedicines10051132 (PMC9139038; doi:10.3390/biomedicines10051132)
Supplement: Supplementary file 1 [file biomedicines-10-01132-s001.zip › biomedicines-1672183-supplementary.pdf]

Supplementary Tables

Table S1: The MNI coordinates and anatomical labels of each NIRS channel

Table S2: Correlation table showing the relationship between age, reaction time measures and fNIRS functional connectivity measures

Table S3: Correlation tables between WCST reaction time and PFC FC parameters (subgroup analyses)

Table S1: The MNI coordinates and anatomical labels of each NIRS channel

Channel number	x	y	z	Anatomical labels (listed by desending % of overlap)
CH01	−75.1	−38.7	114.7	SupraMarginal_R
CH02	−71.15	−62.05	101.1	Postcentral_R, Precentral_R
CH03	−60.1	−83.5	90.9	Frontal_Inf_Tri_R, Frontal_Inf_Oper_R, Frontal_Mid_R
CH04	−41.55	−100.75	84.65	Frontal_Mid_R
CH05	−17.55	−109.8	81.3	Frontal_Sup_R, Frontal_Mid_R, Frontal_Sup_Medial_R
CH06	10.4	−109.95	80.45	Frontal_Sup_L, Frontal_Sup_Medial_L
CH07	40.7	−98.85	83.3	Frontal_Mid_L
CH08	62.35	−80.2	89.3	Frontal_Inf_Tri_L, Frontal_Inf_Oper_L
CH09	75.4	−58.7	96.4	Postcentral_L, Precentral_L
CH10	81.5	−34.45	105.8	SupraMarginal_L
CH11	−78.75	−22.3	108.85	SupraMarginal_R
CH12	−79.5	−46.9	92.55	Postcentral_R, SupraMarginal_R
CH13	−71.5	−70.45	80.6	Precentral_R, Frontal_Inf_Oper_R, Frontal_Inf_Tri_R
CH14	−58.35	−92.5	71.4	Frontal_Inf_Tri_R, Frontal_Mid_R
CH15	−33.55	−107.9	65.95	Frontal_Mid_R, Frontal_Sup_R
CH16	−4.95	−112.05	63.95	Frontal_Sup_Medial_R, Frontal_Sup_Medial_L
CH17	27.2	−107.85	64.95	Frontal_Sup_L, Frontal_Mid_L
CH18	55.2	−90	69.25	Frontal_Inf_Tri_L, Frontal_Mid_L
CH19	70.1	−67.7	76.05	Frontal_Inf_Oper_L, Frontal_Inf_Tri_L
CH20	81.3	−43.2	84.65	Postcentral_L
CH21	82.9	−18.75	97.3	Temporal_Sup_L, SupraMarginal_L
CH22	−83.15	−30.5	86.7	Temporal_Sup_R, SupraMarginal_R
CH23	−79.85	−55.3	72.05	Postcentral_R, Rolandic_Oper_R, Precentral_R
CH24	−69.75	−79.45	61.1	Frontal_Inf_Tri_R
CH25	−50.35	−99.65	52.7	Frontal_Mid_R
CH26	−20.95	−110.15	48.6	Frontal_Sup_R, Frontal_Sup_Medial_R
CH27	11.85	−109.95	48.45	Frontal_Sup_L, Frontal_Sup_Medial_L
CH28	41.7	−99	50.9	Frontal_Mid_L
CH29	62.95	−77.5	56	Frontal_Inf_Tri_L
CH30	76	−52.2	64.3	Rolandic_Oper_L, Frontal_Inf_Oper_L, Postcentral_L
CH31	82.7	−27.5	76.15	Temporal_Mid_L, Temporal_Sup_L, SupraMarginal_L
CH32	−82.9	−13.6	79.15	Temporal_Sup_R, Temporal_Mid_R
CH33	−81.95	−38.5	63.85	Temporal_Sup_R
CH34	−74.35	−64.2	51.75	Frontal_Inf_Oper_R, Frontal_Inf_Tri_R, Rolandic_Oper_R
CH35	−62.05	−87.9	41.05	Frontal_Inf_Tri_R, Frontal_Inf_Orb_R
CH36	−37.55	−105.05	33.75	Frontal_Mid_Orb_R, Frontal_Mid_R, Frontal_Sup_Orb_R
CH37	−5.55	−108.55	31.6	Frontal_Mid_Orb_R, Frontal_Mid_Orb_L, Frontal_Sup_Medial_R
CH38	27.25	−105.35	32.95	Frontal_Mid_Orb_L, Frontal_Sup_L, Frontal_Sup_Orb_L
CH39	54.15	−86.9	36.95	Frontal_Inf_Orb_L, Frontal_Inf_Tri_L, Frontal_Mid_Orb_L
CH40	68.7	−60.65	44.85	Temporal_Pole_Sup_L, Frontal_Inf_Tri_L
CH41	79.55	−37	54.2	Temporal_Mid_L
CH42	84.4	−10.9	66.75	Temporal_Mid_L

CH43	−81.7	−21.6	56.3	Temporal_Mid_R, Temporal_Sup_R
CH44	−76.45	−47.4	43.55	Temporal_Sup_R, Temporal_Mid_R
CH45	−66.65	−72.65	31.7	Frontal_Inf_Orb_R, Frontal_Inf_Tri_R
CH46	−49.25	−93.3	22.1	Frontal_Mid_Orb_R, Frontal_Inf_Orb_R
CH47	−22.15	−103.45	16.75	Frontal_Sup_Orb_R, Frontal_Mid_Orb_R
CH48	9.85	−103.95	16.1	Frontal_Sup_Orb_L, Frontal_Mid_Orb_L
CH49	39.7	−93.25	19	Frontal_Mid_Orb_L, Frontal_Inf_Orb_L
CH50	59.9	−70.05	25.8	Frontal_Inf_Orb_L
CH51	72.25	−45.45	34.75	Temporal_Mid_L
CH52	81.25	−20.4	44.8	Temporal_Mid_L, Temporal_Inf_L

Table S2: Correlation table showing the relationship between age, reaction time measures and fNIRS functional connectivity measures

Group/ Parameters	RT_acquisition	RT_application	R_lPFC_acquisition	R_lPFC_application	L_lPFC_application
**Whole group**	−0.053	0.046	0.181	0.159	0.171
**TD subgroup**	−0.173	−0.086	0.189	0.066	−0.005
**ASD subgroup**	0.239	0.415	0.139	0.194	0.095

Note: N = 51 (listwise); Spearman's rho correlation analyses were performed.

Table S3: Correlation tables between WCST reaction time and PFC FC parameters (subgroup analyses)

a) TD subgroup

	R_lPFC_acquisition	R_lPFC_application	L_lPFC_application
**RT_acquisition**	−0.397	−0.456	−0.443
**RT_application**	−0.406	−0.529 **	−0.461

Note: N = 24 (listwise); Spearman's rho correlation analyses were performed.

b) ASD subgroup

	R_lPFC_acquisition	R_lPFC_application	L_lPFC_application
**RT_acquisition**	0.022	0.062	0.155
**RT_application**	0.041	−0.018	−0.119

Note: N = 27 (listwise); Spearman's rho correlation analyses were performed.
